# Sirt3, Mitochondrial ROS, Ageing, and Carcinogenesis

**DOI:** 10.3390/ijms12096226

**Published:** 2011-09-23

**Authors:** Seong-Hoon Park, Ozkan Ozden, Haiyan Jiang, Yong I. Cha, J. Daniel Pennington, Nukhet Aykin-Burns, Douglas R. Spitz, David Gius, Hyun-Seok Kim

**Affiliations:** 1 Department of Cancer Biology, Pediatrics, and Radiation Oncology, Vanderbilt University Medical Center, Nashville, TN 37232, USA; E-Mails: romad96@gmail.com (S.-H.P.); ozkan.ozden@vanderbilt.edu (O.O.); haiyan.jiang@vanderbilt.edu (H.J.); yong.cha@vanderbilt.edu (Y.I.C.); daniel.pennington@vanderbilt.edu (J.D.P.); david.gius@vanderbilt.edu (D.G.); 2 Free Radical and Radiation Biology Program, Department of Radiation Oncology, Holden Comprehensive Cancer Center, The University of Iowa, Iowa City, IA 52242, USA; E-Mails: naykinburns@uams.edu (N.A.-B.); douglas-spitz@uiowa.edu (D.R.S.)

**Keywords:** *Sirt3*, mitochondria, acetylation, acetylome, cancer, MnSOD, carcinogenesis, receptor positive breast cancer

## Abstract

One fundamental observation in cancer etiology is that the rate of malignancies in any mammalian population increases exponentially as a function of age, suggesting a mechanistic link between the cellular processes governing longevity and carcinogenesis. In addition, it is well established that aberrations in mitochondrial metabolism, as measured by increased reactive oxygen species (ROS), are observed in both aging and cancer. In this regard, genes that impact upon longevity have recently been characterized in *S. cerevisiae* and *C. elegans*, and the human homologs include the Sirtuin family of protein deacetylases. Interestingly, three of the seven sirtuin proteins are localized into the mitochondria suggesting a connection between the mitochondrial sirtuins, the free radical theory of aging, and carcinogenesis. Based on these results it has been hypothesized that Sirt3 functions as a mitochondrial fidelity protein whose function governs both aging and carcinogenesis by modulating ROS metabolism. Sirt3 has also now been identified as a genomically expressed, mitochondrial localized tumor suppressor and this review will outline potential relationships between mitochondrial ROS/superoxide levels, aging, and cell phenotypes permissive for estrogen and progesterone receptor positive breast carcinogenesis.

## 1. Introduction

Mammalian cells express proteins that protect against endogenous and exogenous forms of genotoxic stresses that induce genomic instability [1–3]. These proteins monitor the integrity of cellular metabolism as well as respond to stressful conditions by activating compensatory pathways [4–6]. An extension of this observation would be that the loss of function or genetic mutation of these fidelity proteins creates a cellular environment that is permissive for the development of tumors [7–10], suggesting that these proteins also function as tumor suppressor genes (TSG) in the context of aging and metabolism [11]. Since it is unlikely that evolutionary pressure selected for proteins in mammalian cells to prevent carcinogenesis, these proteins are more likely fidelity proteins that have evolved over time to protect specific organelles from damage induced by agents that induce genotoxic stress.

Genomic instability, a hallmark of cancer, is thought to be an early event in neoplastic transformation induced by genotoxic agents [12–14]. As such one of the paradigms in biology is that mammalian cells contain fidelity proteins that recognize DNA damage and subsequently initiate signaling cascades that maintain cellular processes to prevent the accumulation of genomic damage [1,15,16]. In this context the best known example of a tumor suppressor (TS) gene is p53, which senses cellular damage from multiple endogenous and exogenous genotoxic stresses, including ionizing radiation (IR), and induces reparative processes that prevent IR-induced genomic instability and carcinogenesis [10,17,18]. Thus, it has been proposed that the genomic instability contributes to cellular phenotypes that represent early events in the multi-step genetic, epigenetic, and metabolic processes contributing to carcinogenesis.

Mitochondria may also contain specific fidelity proteins that sense organelle conditions and activate signaling pathways to maintain mitochondrial homeostatic poise [19,20]. One of the primary functions of the mitochondria is the generation of ATP and complex aerobic organisms utilize oxygen as an electron acceptor in respiration, as an extremely efficient method of generating ATP [13]. However, by-products of mitochondrial electron transfer reactions in aerobic cells result in the production of ROS (*i.e*., superoxide and hydrogen peroxide) from the incomplete reduction of dioxygen [21]. The accumulation of abnormally high levels of mitochondrial superoxide from any source can create a condition referred to as “oxidative stress” that damages cells and is thought to contribute to both carcinogenesis and aging [22,23]. While low steady-state levels of ROS are easily tolerated and perform necessary signaling functions to coordinate metabolic and genetic processes [13], abnormally high levels of ROS from any number of possible sources can damage cells [24,25] and create a cellular environment permissive for genomic instability as well as carcinogenesis [2]. Thus, it has been proposed that mitochondrial damage, from any of a number of causes, might result in increased ROS that are causative agents in the development of genomic instability [26–28].

## 2. Sirtuins as Fidelity or Tumor Suppressor Proteins

Sirtuins are NAD^+^ dependent class III histone deacetylases which are present from bacteria to humans [29,30]. Unlike traditional histone deacetylases, sirtuins dynamically deacetylate a variety of substrates ranging from transcription factors to metabolic enzymes as well as histones [31,32]. In addition, sirtuins require NAD^+^ as a co-factor which makes them a metabolic sensor and connects their enzymatic activity to the energy and redox state of cells [33,34]. One theme that has emerged in the last several years is that aging, perhaps better defined as longevity, is a complex cellular process that appears to be regulated, at least in part, by a relatively new gene family referred to as sirtuins. Sirtuin genes are the human and murine homologs of the *Saccharomyces cerevisiae Sir2* gene that has been shown to regulate both replicative and overall lifespan [3,4]. The sirtuin family genes also regulate longevity in *C. elegans* and *D. melanogaster* [35,36].

In lower organisms, sir2 plays a role in longevity by enhancing multiple mechanisms including but not limited to silencing of telomeres and sub-telomeric regions, silent mating type loci, and, crucially, the rDNA, suppressing formation of rDNA circles [36,37]. On the other hand, mammalian sirtuin members are associated with numerous physiological roles, such as stress response, regulation of metabolism, gene silencing, and aging [38,39]. While it has not been shown that these genes determine longevity in mammals they do appear to regulate critical signaling networks, and following stress, several mice lacking one of the sirtuin genes develop illnesses that mimic those observed in older humans [36,40]. Thus, it has been proposed that the mammalian sirtuins play a significant role, at least in part, in directing the acetylome signaling network that has recently been shown to be critical in the regulating multiple cellular processes [41]. These proteins share a common 275-amino acid catalytic domain and are localized to the nucleus (SIRT1, 6, and 7), mitochondria (SIRT3, 4, and 5), and cytoplasm (SIRT2), respectively [42]. Unlike histone deacetyl transferase the sirtuins primarily target cellular proteins other than histones suggesting these proteins are critical the regulation of cell signaling networks similar to phosphatases and kinases [39,41]. Sirtuins therefore appear to function as signaling proteins that post-translationally alter the activity of downstream protein targets via acetylation.

## 3. Sirtuins Monitor and Direct Cellular Metabolism and Stress

Sirtuin activity can be increased in response to metabolic, genotoxic, oxidative, and osmotic stresses. These stress responses appear to link to aging [43–45], oxidative stress, and the free-radical theory of aging (FRTA) [46]. In this model it is proposed that there is a mechanistic connection between aberrant cellular ROS levels and aging. Thus, it was proposed that an organism ages due to the unrepaired accumulation free radical damage to critical biomolecules as a function of time [1,13]. For most biological structures, free radical damage is closely associated with oxidative damage [47,48]. Thus, this theory would predict that antioxidants or reducing agents, may limit oxidative damage to biological structures by detoxifying free radicals and preventing aging and age-related human illness [21,49,50].

Sirtuins, including the mitochondrial sirtuin Sirt3, appear to respond to changes in cellular and nutrient stress resulting in the activation of their deacetylase or ribosyltransferase activity resulting in a post-translational modification of downstream target proteins [20,50–53]. For example, it is now well established that Sirt3 deacetylation activity is activated by caloric restriction (CR) and fasting [20,50,53] and this induction in deacetylation activity also appears to protect against the development of age-related human pathology including cancer induction [20,54]. While the mice lacking *Sirt3* do not exhibit changes in life span it has been shown in human studies that *SIRT3* expression levels has been found to be associated with higher longevity [55]. In this regard, CR is the most effective non-genetic intervention described to increase lifespan across a wide variety of phyla [56]. While this phenomenon has been appreciated for many decades, the exact mechanism(s) remain unclear. While this effect has not been directly demonstrated in humans, humans may undergo many of the same metabolic changes and experience the same health effects in response to CR as seen in lower organisms [57]. In fact, it can be argued that CR may be the single best method identified to decrease the incidence of developing cancer and CR clearly decreases intracellular ROS [58]. Since it is now well established that CR activates sirtuins, including Sirt3, it seems reasonable to suggest a potential connection between sirtuins, cellular metabolism as measured by aberrant mitochondrial ROS, and age-related illness and pathologies including carcinogenesis.

## 4. Sirtuins and Regulation of the Mitochondrial Acetylome

The sirtuins appear to play a central role, at least in part, in regulating the cellular acetylome responding to endogenous and exogenous cell stress and nutrient distress [39,59]. Thus, is has been proposed that at least one function of the sirtuin gene family is the regulation and maintenance of the Metabolome via the deacetylation of specific downstream target proteins that direct intracellular oxidation/reduction pathways [37,41]. Reversible acetylation of lysine is a post-translational modification that neutralizes the positive charge of this amino acid, potentially altering the 3-dimensional structure of a protein as well as its enzymatic function [60,61]. One well known example of this is the histone deacetylases (HDACs) and histone acetyltransferases (HATs) that regulate gene expression through the modification of histone and changes in chromatin remodeling [62].

In this regard lysine acetylation has recently emerged as an important, and perhaps the primary, post-translational modification employed to regulate mitochondrial proteins [63,64]. Several proteomic surveys have identified a disproportionately high number of acetylated proteins in the mitochondria and many of these proteins are associated with energy homeostasis [64,65]. Reversible acetylation of lysines that alter protein structure is mediated by histone deacetylases (HDACs) and histone acetyltransferases (HATs) that regulate gene expression through the modification of histones [62]. Lysine acetylation is also involved in the regulation of p53 which appears to also play a role in mitochondrial redox regulation [66].

Sirt3 is the primary mitochondrial deacetylase and genetic knockout of Sirt3 alter mitochondrial protein acetylation [67] including aberrant/decreased mitochondrial ATP levels *in vitro* and *in vivo* [68]. Based on these results, acetylation of mitochondrial proteins may play a role in maintaining and regulating mitochondrial metabolism and function. Thus, we believe it is a logical extension to hypothesize that Sirt3 is a regulatory protein, maintaining mitochondrial homeostasis via changes in the acetylation of metabolic target proteins. This idea would mechanistically connect a metabolic sensing and/or signaling protein, such as Sirt3, to the direct regulation of mitochondrial energy metabolism, ATP synthesis, detoxification of mitochondrial ROS, as well as other biological processes essential for proper mitochondrial function.

## 5. Sirtuins, Mitochondrial ROS, and Carcinogenesis

Since the rate of malignancies increases significantly as a function of age, several reports have suggested a potential mechanistic link between the cellular process governing longevity and the development of cancers [58,69]. In addition, it is well established that the mitochondria of tumor cells exhibit aberrant ROS and this observation has been suggested to account for the high degree of genomic instability demonstrated in cancer cells [46,70]. It is also a fundamental hypothesis in cancer research that increased or aberrant cellular mitochondrial ROS is an early event in cell damage that results in the generation of genomic stability that, under specific cellular conditions, can results in dedifferentiation and carcinogenesis [12,13,71]. These observations suggest a potential mechanistic connection between mitochondrial function and carcinogenesis; however, until recently there were a lack of genetic models to investigate this hypothesis.

Sirt3 appears to be the primary mitochondrial deacetylase it has been proposed that Sirt3 may function as a mitochondrial fidelity gene [72,73]. We hypothesized that critical regulators at the crossroads between aging and loss of Sirt3 deacetylase activity may create a cellular environment permissive for age-related cancers [19]. Thus, six years ago we, and others, constructed mice lacking the mitochondrial Sirt3 gene to determine if Sirt3 is a fidelity gene and if loss of function would create an *in vivo* tumor permissive phenotype.

Sirt3 is the mitochondrial matrix protein that is present in high amount in mouse kidney, heart, liver, and brown adipose tissues. Mice lacking *Sirt3* do not display any significant observable acute physiological abnormalities; however, they have a large number of acetylated proteins in their mitochondria relative to wild-type mice [67,73]. However, when these animals or their cultured cells were challenged with various stress factors, such as oxidative stressors, chemical-hormonal, or ionizing radiation, they displayed physiological phenotypes consistent with increasing age. These phenotypes included cardiac hypertrophy [51,74], carcinogenesis [20,75,76], fatty liver [52,77], radiation-induced liver damage [50], and age-related hearing loss [54,78]. While it is unlikely that there is one all encompassing, overarching mechanism that underlie above-mentioned *in vivo* phenotypes, each of these publications identified increased cellular ROS as a potential underlying etiology and suggest a potential connection between the development of these pathologies and aberrant mitochondrial metabolism. Therefore these results support the hypothesis that excessive levels of ROS is one mechanism of shortened life span and age-associated pathological conditions, as suggested by the free radical theory of aging [46,70].

## 6. SIRT3 Is a Genomically Expressed, Mitochondrial Localized TS Protein

In this regard, Sirt3 was reported to protect cells against ROS by enhancing the activity of antioxidant defense system, suggesting that this protein could be a crucial mitochondrial fidelity protein in response to oxidative stress [20,50,53,54]. Whereas Sirt3 deficiency was well tolerated in young mice in normal conditions, at ages greater than one year old *Sirt3* knockout mice exhibited a deterioration liver mitochondrial DNA, lower ATP production, increase mitochondrial ROS including superoxide levels, and developed ER/PR positive breast malignancies [20]. These results suggested that Sirt3 might function as a mitochondrial fidelity protein and loss of function of Sirt3 may establish cellular environment permissive for aberrant mitochondrial metabolism, oxidative stress, and carcinogenesis.

In this regard, *Sirt3* knockout mouse embryonic fibroblasts (MEFs) exposed to various stress factors, such as oxidative, genotoxic stress, and radiation exposures resulted in a loss of contact inhibition. The Sirt3^−/−^ MEFs were also immortalized and transformed by infection with a single oncogene suggesting that Sirt3 might function as an *in vitro* tumor suppressor. These results were confirmed when mice lacking Sirt3 spontaneously developed mammary tumors appearing at just over one year of age. Finally, the interrogation of human breast cancer samples as measured via: (1) staining for protein levels; (2) RT-PCR for RNA levels; and (3) genomic analysis all showed a statistically significant decrease in SIRT3 levels in tumor cells, as compared to normal tissue controls. As such, we would propose that these results very strongly suggest that SIRT3 is a genomically expressed, mitochondrially localized tumor suppressor protein [20]. In this regard, the Sirt3 knockout mice do not begin to develop mammary tumors until after 12 months of age the immunohistochemical staining has identified these tumors as well differentiated estrogen (ER) and progesterone (PR) receptor positive mammary tumors [20]. This result seems logical since these mice lack a gene that has been suggested to play a role in aging or, perhaps better stated, anti-aging, and receptor positive breast malignancies are most common in post-menopausal women and have a very strong statistically correlation with increasing age [58,69]. In fact breast cancer has a very specific age-related incidence that increases slowly until the mid 60’s when a significant increase in incidence is observed [58].

While our work [20], and another rigorously done manuscript [75,76], have both shown that Sirt3 functions as a TS, the detailed mechanism linking Sirt3-mediated mitochondrial ROS and carcinogenesis still remains to be fully determined. In this regard, the subcellular localization of Sirt3 is in close proximity to the main source of ROS production and this renders Sirt3 in the ideal location for the protection from aging and age-associated disorders including carcinogenesis. Elevated mitochondrial oxidative stress was proposed to play a role, at least in part, for the development of breast cancers in humans [79,80] as well as the development of mammary tumors in the aged *Sirt3* knockout mice. Consistent with this data, human breast tissue samples also displayed increased mitochondrial superoxide levels and decreased Sirt3 expression and the Sirt3^−/−^ mouse hepatocytes [20] and cardiomyoctes [51,74] also showed significantly higher basal superoxide levels that were observed to further increase when exposed to different types of exogenous cellular stress challenges. These results suggested, but did not prove, a direct connection between aberrant mitochondrial superoxide levels and the tumor permissive phenotype observed in mice and MEFs lacking Sirt3.

A more convincing argument for the role of aberrant mitochondrial superoxide levels in cells lacking Sirt3 was shown using duel infection experiments with lentivirus expressing MnSOD. MnSOD is the primary superoxide detoxification enzyme that converts superoxide to hydrogen peroxide that is finally converted to water by catalase [81]. In these tissue culture experiments, co-infection of lenti-MnSOD not only decreased mitochondrial superoxide levels but also prevented immortalization of Sirt3^−/−^ MEFs by a single oncogene [20]. These experiments were confirmed using a MnSOD gene where lysine 122 was mutated to an arginine, MnSOD^122K-R^, resulting in a constitutively active dominant positive MnSOD protein [50]. Co-infection of lenti-MnSOD^122K-R^ also prevented immortalization of Sirt3^−/−^ MEFs by a single oncogene. In contrast, co-infection with mutated dominant negative MnSOD gene, lenti-MnSOD^122K-Q^, mimicking a constitutively acetylated lysine, failed to prevent immortalization by infection with a single oncogene [50]. Similar experiments have shown that MnSOD lysine 68 [82] and lysines 53 and 89 [53] also appear to be deacetylated by SIRT3 as well as alter MnSOD enzymatic activity. Finally, it has also shown that infection of a MnSOD dominant positive (lenti-MnSOD^122K-R^) gene prevented tissue culture transformation with exogenous agents such as ionizing radiation as well as stress-induced increases in cellular ROS [50]. All these experiments strongly suggest that aberrant mitochondrial superoxide metabolism, at least in part, plays a significant role in the tumor permissive phenotype observed in cells lacking Sirt3.

**Figure 1:**
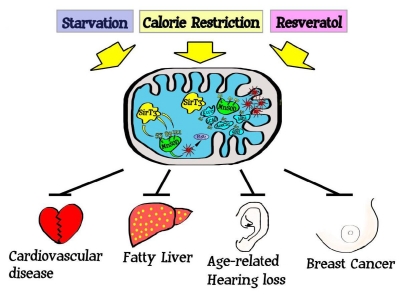
Proposed model describing Sirt3 acetylation and subsequent regulation of downstream target proteins enzymatic activity. Sirt3 is localized into the inner mitochondrial membrane and appears to be activated by agents that induce oxidative stress and respond to aberrant or increased mitochondrial levels of superoxide. Sirt3 has been shown to regulate the activity of other mitochondrial proteins including acetyl-CoA synthetase [63,84], glutamate dehydrogenase (GDH) [84,85], long-chain acyl-CoA dehydrogenase (LCAD) [46], succinate dehydrogenase [86,87], and mitochondrial ribosome subunit MRPL10 [87,88]. Sirt3 has also be shown to have pro-apoptotic or anti-apoptotic effects on different cell types and at least on mechanism involves deacetylating Ku70 and consequently preventing the release of BAX into mitochondria [88,89].

While this work, as well as that recently published from the Guarente and Haigis laboratories [75,76,83], suggests a mechanistic connection, at least in part, between aberrant mitochondrial superoxide metabolism and carcinogenesis it seems like factors other, such as HIF-1α than metabolism are involved. For example, we now know that a variety of important metabolic and cell survival related mitochondrial targets of Sirt3 including acetyl-CoA synthetase [63,84], glutamate dehydrogenase (GDH) [84,85], long-chain acyl-CoA dehydrogenase (LCAD) [46], succinate dehydrogenase [86] and mitochondrial ribosome subunit MRPL10 [87]. Sirt3 has also be shown to have pro-apoptotic or anti-apoptotic effects on different cell types and at least on mechanism involves deacetylating Ku70 and consequently preventing the release of BAX into mitochondria [88]. Finally, several manuscripts have identified multiple mitochondrial proteins that contain proteins (Figure 1) with reversible acetyl lysine(s) and many of these have been shown to be altered in cancers [64,65,67]. Thus, similar to p53, aberrant regulation of several proteins in the mitochondrial acetylome may play some role in the cellular damage and tumor permissive phenotype observed in mice lacking *Sirt3*.

One final question is how aberrant regulation of the mitochondrial acetylome, results in spontaneous, stress-induced genomic instability and can loss of proteins other than Sir3 result in spontaneous genomic instability [1]. In this regard, a somewhat similar finding was recently reported in *Saccharomyces cerevisiae* by the Gottschling laboratory [90]. In this work it was shown that a reduction in the mitochondrial membrane potential resulted in a cellular crisis identified via a defect in iron-sulfur cluster (ISC) biogenesis, which is required for normal mitochondrial function. This crisis resulted in downregulation of ISC protein biogenesis causing genomic instability, suggesting mitochondrial dysfunction stimulates nuclear genome instability by inhibiting the production of ISC-containing proteins.

Our results also suggest that increased mitochondrial superoxide levels, which exceed the ability of MnSOD to detoxify superoxide, may escape the mitochondria and subsequently increase both the cytoplasmic and nuclear superoxide levels. However, this idea is controversial since many researchers believe that superoxide cannot move out of the mitochondria. Thus, can superoxide generated in mitochondria enter the cytosol via passage through voltage dependent or independent channels in the mitochondrial membranes causing alterations in redox signaling in the cytosol, potentially contributing to genomic instability in the nucleus? The evidence supporting this hypothesis is mostly indirect using anion channel blockers such as DIDS (4,4′-diisothiocyanatostilbene-2,2′-disulfonate) [91,92] or other methods [93]. While this review cannot answer this controversy, at least one mechanism for the increased genomic instability in mice and MEFs lacking *Sirt3* appears to involve increased mitochondrial superoxide levels that use either existing ISC or other membrane channels to escape into cellular organelles outside of the mitochondria.

## 7. Conclusions

One fundamental observation in eukaryotic biology is that mammalian cells contain fidelity proteins that function to monitor the integrity of critical intracellular processes, and deletion or mutation of the corresponding genes results in a cellular environment permissive for the accumulation of damage [10,11,94]. Thus, it seems like a logical extension that the mitochondria would also contain fidelity proteins to maintain the integrity of the mitochondria. In this regard, loss of Sirt3 results in a series of stress and/or aging-related murine phenotypes including receptor positive breast cancer [20], fatty liver [52,77], insulin resistance [52], cardiac hypertrophy [51,74], radiation-induced liver steatosis [50], and age-associated hearing loss [54]. While it may be premature to make a cause and effect connection between age-related pathologies and alteration in mitochondrial ROS it seems likely that aberrant mitochondrial ROS plays a role, at least in part, in these murine phenotypes. Finally, it also seems clear that Sirt3 is a mitochondrial localized tumor suppressor or fidelity protein and identifying the downstream deacetylation targets of Sirt3 will lead to better understanding of mechanism linking mitochondrial fidelity, carcinogenesis, and aging.
